# Mitigating Climate Impacts of “Hygiene Theatre” in Health Care: Perspectives of Primary Health Care Providers in Ontario, Canada

**DOI:** 10.3390/healthcare14131921

**Published:** 2026-07-01

**Authors:** Paul Gregory, Zubin Austin

**Affiliations:** Leslie Dan Faculty of Pharmacy, University of Toronto, Toronto, ON M5S 1A1, Canada; paul.gregory@utoronto.ca

**Keywords:** hygiene theatre, primary health care, climate impact mitigation

## Abstract

**Highlights:**

**What are the main findings?**

- Hygiene theatre practices in health care work persist due to the limited ability of professionals to self-reflect and critically examine their own practices.
- Reducing unnecessary hygiene theatre will require significant education of health care professionals and patients, with a focus on cost savings and the absence of clinical evidence rather than climate mitigation impacts alone.

**What are the implications of the main findings?**

- Reliance on the self-reflection of clinical practices as a primary pathway to reduce unnecessary hygiene theatre is unlikely to be successful.
- Collaboration, education, guidance, and regulation from external groups (including regulators, employers, educators, professional associations, and unions) will be required in order to shift hygiene practices to an evidence-based foundation with positive climate mitigation impacts.

**Abstract:**

**Background/Objectives:** Hygiene theatre describes a diverse array of cleaning and sanitation protocols (such as the use of disinfectant sprays, or plexiglass dividers) that may provide a false sense of safety/security without actually or meaningfully reducing the risk of transmission of pathogens. Initially viewed as a humorous, but harmless, contrivance, the carbon footprint implications and climate impacts of unnecessary and unhelpful performative clinical activities is increasingly being scrutinized. This study examined primary health care providers’ perspectives on hygiene theatre and how to mitigate or reduce both its prevalence and its impact. **Methods:** Semi-structured interviews with 17 family physicians, nurses, nurse practitioners, and pharmacists were conducted. **Results:** The findings suggest that pervasive and persistent hygiene theatrics may reflect primary care providers’ inability to critically self-reflect on routinized clinical practices due to a lack of time, the inaccessibility of clinical evidence, and a lack of workplace supports. **Conclusions:** Addressing hygiene theatre may benefit from direction, guidance or regulation from external groups such as employers, unions, or licensing bodies. The further education of patients (who may have come to expect these theatrics) may also be necessary to better manage their expectations.

## 1. Introduction

Cleanliness and sterility are central to health care work, ever since the “germ theory” of medicine became widely accepted [1]. The notion that disease-causing pathogens can spread illness within a family, community, society—or a health care setting like a hospital or primary care practice—is well accepted by health care professionals and the public. Reducing the risk of transmission of preventable infectious diseases through careful and routine cleaning practices is integral to operations in all health care facilities, and a baseline expectation of practitioners and patients alike [1,2]. Activities ranging from surface decontamination with disinfectant/antimicrobial solutions to the installation of plexiglass barriers to the universal use of alcohol swabs prior to vaccination have become commonplace, in the name of reducing the risks associated with the spread of infectious diseases [2].

Differentiating necessary and impactful infection control procedures from those that may be less valuable requires high-quality evidence. Since the COVID-19 pandemic, questions have emerged regarding the threshold of evidence that defines prudent and defensible infection control practices [2]. While public health officials are typically the most valued/trusted source of information with respect to infection control practices, some common activities (for example, the use of plexiglass shields at office reception desks) may not be widely endorsed by public health officials due to mixed or inconclusive evidence as to their value in preventing the spread of infections [2]. As a result, there may be concerns that certain infection control practices—particularly those that emerged rapidly and with limited or mixed evidence during pandemic conditions—continue to be perpetuated.

The term “hygiene theatre” was likely first coined by blogger Bob Cooney (a virtual reality consultant) in early March 2020 as the COVID-19 pandemic first began [2]. The term was presented as a customer-service practice designed to reassure patrons at virtual-reality arcades that they were safe because the equipment used in these places had been cleaned to a higher degree of sanitation than was required or expected. Derek Thompson, a staff writer for *The Atlantic*, further popularized the use of this term in an article referring to COVID-19 cleanliness practices that had, in reality, done little to reduce the spread of the virus, and as a result, had imparted a false sense of security to the general public [3].

“Hygiene theatre” as a term initially provoked some mild bemusement, particularly in the context of a life-changing worldwide global pandemic where scientific understanding of the causative agent (SARS-CoV-2) was lacking, evidence was rapidly evolving, and guidance around safety protocols was constantly changing. The inclusion of “theatre” in the term reflected the uncomfortable perspective that the elaborate rituals associated with pandemic protection were simply performative, and not actually helpful in reducing the risks of transmission. Still, in a time of uncertainty and fear, performative rituals were thought to have positive emotional benefits that outweighed the costs in terms of supplies/materials, the staff time required to deploy them, and the carbon impacts associated with the unnecessary production and use of hygiene-related materials [4].

As pandemic conditions subsided and public and professional life reverted to pre-pandemic norms, many COVID-19-era hygiene practices have persisted. Pre-pandemic practices and assumptions around cleanliness and sterility have also persisted and in some cases expanded in lockstep with COVID-19-era practices, particularly in the context of health care work and within health care facilities such as hospitals, primary care clinics, and pharmacies. Examples of these practices include:

(A) Deep cleaning (sanitizing): The abundant use of disposable antimicrobial-infused wipes and sprays to “deep clean” surfaces is ubiquitous. These products purport to leverage antimicrobial chemicals to arrest growth or kill potentially harmful infectious pathogens that could potentially spread when human hands touch surfaces such as counters or doorknobs.
(B) Plexiglass barriers: Physical barriers designed to prevent the spread of aerosolized particles released from the mouth and nose do so at the expense of interpersonal communication, including impacts on audibility and impaired interpretation of non-verbal communication cues related to empathy.
(C) Temperature checking: Many infections present with increases in body temperature; a fever is often an early or mid-course marker of a bacterial or viral pathogen. Temperature checking was commonplace during the pandemic and continues in some health care settings. The mass surveillance of temperature usually requires the use of disposable cleaning supplies or probe tips.
(D) Alcohol swabbing: Many vaccination protocols require or endorse the use of disposable alcohol swabs used at the injection site, for the stated purpose of reducing the risk of infection during the vaccination process itself. When one considers the (literally) billions of COVID-19 vaccines administered, all of which were accompanied by tiny foil packages and alcohol- or antimicrobial-infused napkins that ended up in the garbage or landfill, the climate impact of this single intervention is monumental.
(E) Single-use/unit-of-use medical and pharmaceutical supplies: The use of disposable, single-use items in health care work is ubiquitous and justified on the grounds that it reduces the risk of contamination and the transmission of infectious diseases. The packaging associated with single-use products can be significant (for example, medication blister packs where a single table designed for a single patient to use involves foil and plastic coverage, as opposed to removing a single table from a large stock bottle containing 500 tablets or more).
(F) Hand cleaning: Since the pandemic, bottles of hand sanitizer at doorways and entrances have become commonplace and widely used. Such sanitizer products are complex biochemically and may be minimally or negligibly biodegradable, despite being used on human hands.

Critics of practices such as these note that they distract attention from more expensive, but possibly more impactful, changes such as enhanced ventilation or air quality systems that address airborne transmission vectors favoured by many pathogens [4,5]. Other practices—such as the use of personal protective equipment (such as well-fitting high-quality face masks or latex gloves)—may also be considered “theatrics” when they are incorrectly deployed by human users, or used in circumstances where they are neither necessary nor beneficial [6].

A central feature of hygiene theatre interventions involves single-use disposal items in order to minimize the risk of contamination and pathogen spread. The carbon impacts of single-use disposable items (including plastic straws and cutlery) have been vigorously debated in other parts of society, yet there has been relatively scant attention paid to this topic within health care [7]. Another feature of hygiene theatre is its reliance on chemical antimicrobials as a tool to arrest growth or kill pathogens on surfaces (including hands). The environmental implications of the use of antimicrobials, including the impacts with respect to the growing antimicrobial resistance associated with such widespread and casual use, have not been fully evaluated [8].

Patients and health care professionals understand and value cleanliness, but the dimensions of antimicrobial or pathogen sterility that are the “promise” of hygiene theatre are less well understood. For example, the incremental benefit associated with wearing gloves or using hand sanitizer vs. washing hands with soap/water rarely trigger discussion, debate or self-reflection on clinical practice [9]. Instead, busy clinicians may simply do what others around them do and follow whatever protocols they are given. Understandably, overly cautious (though wasteful) practices that are supposed to enhance cleanliness and reduce the risk of the transmission of pathogens are not the most obvious first choice for busy clinicians to critique. As a result, the climate impacts of unnecessary, unhelpful, and performative hygiene theatre may not be an object of clinical self-improvement or quality assurance, and a significant opportunity to mitigate health-care-related climate harms may go unchallenged. A central tension exists with respect to the evidence of the value and infection-mitigation impact of certain practices. It is difficult for many clinicians to differentiate between evidence-based infection prevention interventions and other practices where limited or questionable evidence exists within specific settings [1]. In such a context, there may be a tendency to assume that it is safer to overcompensate with hygiene theatrics [1], without fully assessing the climate-related impacts that may result.

The objective of this research was to characterize the perspectives of primary health care professionals (including family physicians (general practitioners), nurses, nurse practitioners and pharmacists) with respect to hygiene theatre practices and to elicit their points of view on how best to mitigate the climate-related impacts of these practices in ways that minimize an increased risk of the spread of pathogens, in the context of enhancing opportunities for climate-conscious primary care practice.

## 2. Materials and Methods

The concept of hygiene theatre is relatively new, and as a result, there is minimal available literature or research examining health professionals’ perspectives and opinions on this topic. Thus, an exploratory research method focused on qualitative approaches was identified as the most appropriate to begin the exploration of this evolving area of research interest, as a first step in identifying future directions and opportunities for research [10]. Importantly, the exploratory nature of this research was not focused on the hygiene practices themselves; these were selected a priori as examples of clinical activities for which mixed or inconclusive evidence of the infection-control or prevention value existed. The exploratory element of this study focused on the clinical perspectives and experiences of the participants themselves, as this was a topic of research that has not been previously reported in the literature.

Semi-structured interviews were selected as the most appropriate qualitative research method. This method allows a general structure to be rapidly adapted in real time to adjust to an individual research participant’s experiences and perspectives [11]. Semi-structured interviews use a general protocol of questions to provide some direction and a perimeter to the interview (to ensure that it conforms with a central research objective), but they also allow for conversational flexibility to guide the interview, allowing for expansive opportunities to explore new topic areas and evolving areas of research interest.

**Figure 1:**
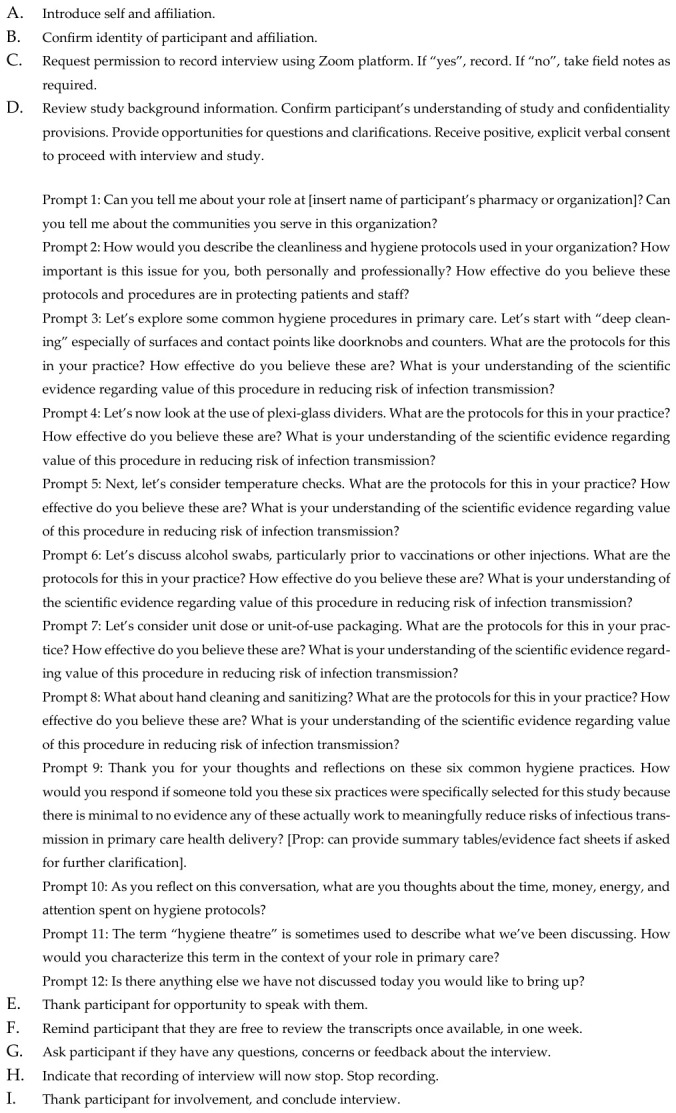
Semi-structured interview protocol (final version, following pilot testing and modifications).

For this research, a semi-structured interview protocol was initially developed that focused the attention of research participants on the concept of hygiene theatre, and to six common hygiene theatre practices encountered in primary care: deep cleaning (sanitization), plexiglass barriers, temperature checking, alcohol swabbing, single-use medical/pharmaceutical supplies, and hand sanitizing using antimicrobial liquids (as opposed to simple hand washing). For the purposes of this study, the use of personal protective equipment (PPE) such as face masks was not defined as hygiene theatre, as sufficient evidence exists regarding its value when it is used/installed correctly by the human user under appropriate/rationale circumstances. The initial draft of the interview protocol provided opportunities for the interviewer to discuss these practices with the participant, allowing the participant to discuss their own clinical experience and behaviours associated with that particular practice. The intent was not to challenge or debate with the participant in the context of the available evidence associated with the practice, but instead to learn more from participants about how these practices were applied in their primary care settings. The initial version of the protocol was pilot tested with four volunteers, and revisions were made based on this testing to enhance the usability, comprehensibility, and efficiency of the protocol. For example, including both the trade names and generic names of medications (rather than simply generic names) was found to be helpful and incorporated into the protocol, as some primary care practitioners are less familiar with generic names (e.g., “acetaminophen”) than trade names (e.g., “Tylenol”). Further minor revisions to the protocol were made during the course of actual interviews, following interviews # 3 and #5, to enhance the efficiency of the interview process. For example, providing the interviewer with greater flexibility in skipping certain questions that may have already been answered through a previous question/discussion was formally integrated into the protocol to enhance the time efficiency of the process. The final version of the semi-structured interview protocol is presented in Figure 1.

Given the busy lives of most health care professionals, the study was designed so that the interviews could be conducted virtually, to minimize inconvenience for the participants. The Zoom platform was selected, as it is widely used and facilitates both recording and the initial transcription of interviews into text form. All the initial transcription texts were carefully reviewed by the interviewer and “cleaned” (i.e., adjusted) by the interviewer to ensure that accurate transcriptions were captured. Adjustments typically involved the clarification of homonyms that were incorrectly transcribed by Zoom (e.g., “sight” instead of “site” or “cite”), sound-alike terms that were incorrectly captured (e.g., “take” vs. “make”) or complex medical terminology or pharmaceutical produce names (e.g., “providing” vs. “poviodone”). These transcripts were then incorporated as the dataset for the analysis, using NVivo v15, a widely used qualitative data analysis software program. NVivo offers the use of artificial-intelligence-driven plug-ins that can be used to summarize transcripts and offer initial coding themes to expedite data analyses. This AI was initially used to provide summaries of the interviews to allow researchers to more efficiently differentiate amongst transcripts, and it provided a more efficient mechanism for establishing the frequency of the use of certain terms or words rather than manual word counting. It also facilitated comparisons between transcripts to highlight situations where similar concepts were described by different research participants using slightly different terminology or words. The AI plug-ins also allowed researchers to examine and code transcripts more efficiently when synonyms (e.g., “used” and “utilized”) were used by participants, rather than relying on manual disambiguation techniques. This descriptive AI technology was used in the research in a human-in-the-loop fashion: all AI-generated outputs from NVivo were reviewed by both researchers independently to ensure that they conformed to the research objectives and the approved research protocol and were adjusted/adapted as required to ensure such conformity.

A constant comparative data analysis method was selected for use in this project. In this method, each researcher independently reviews transcripts, and AI-generated suggestions for coding and categorization for each transcript are reviewed prior to making independent decisions regarding transcript themes [12]. The reviewers would then meet after reviewing two transcripts to compare their analysis, themes and codes and arrive at a consensus. Based on this consensus, a coding tree was generated to guide future analyses and was refined with each meeting of independent reviewers. A consensus between reviewers was required in order to advance the analysis and was facilitated through frequent meetings and grounding in the transcript data itself.

Interviews, data collection, and analyses would continue until the point of thematic saturation, defined as the point at which neither reviewer identified any substantively new themes, codes, or categories in the transcript data [13]. To confirm, two additional interviews following the declaration of thematic saturation were undertaken and an analysis was completed to ensure that saturation had occurred. To ensure quality research, the consolidated criteria for reporting of qualitative research (COREQ) checklist was used to ensure that the study was aligned with best practices to minimize investigator bias and ensure transparency, reproducibility, and quality [14].

The participants in this study were recruited through pre-existing networks of primary care professionals who had agreed to be involved in research such as this. These individuals had agreed to be contacted by email by university-based researchers interested in their perspectives. The introductory email outlined the objectives and methods of the research study and invited those interested in learning more to contact the research team. Those who contacted the research team were then invited to participate in a telephone or Zoom call to learn further details and confirm their interest in participating in this study through the completion of an informed consent process. The informed consent process highlighted the participant’s rights with respect to withholding data, suspending participation in the study at any time without question or risk, and the opportunity to review their personal transcript prior to inclusion in the data analysis. Upon completion of the informed consent, a convenient time for the semi-structured interview was established, and, with permission, it was recorded and transcripts were produced. Since limited funding was available for this work, there was no honorarium or other compensation provided to research participants. Within one week of the completion of their interviews, the participants were provided with an opportunity to review and comment upon their transcripts prior to inclusion in the dataset for analysis.

This research was approved by the institutional research ethics board (protocol 46178, approved 19 May 2025), and was deemed “low risk” research qualifying for expedited review based on (a) the topic area of the research and (b) the nature of the research participants as highly educated professionals. There was minimal risk to the participants involved in this research.

## 3. Results

In response to an initial email invitation to participate in this research, which was sent to 160 primary care professionals (physicians, nurses, nurse practitioners and pharmacists), a total of 31 contacted the research team to learn more about the study and to consider participation. Of these 31, 24 completed informed consent, allowing them to potentially participate. This group was divided into three cohorts: 12 individuals participated in the first round of interviews, with eight participants reserved for round 2 (should it be required, based on attainment of thematic saturation) and four participants reserved for round 3 (should that be required to ensure thematic saturation). Ultimately, the researchers declared thematic saturation after the 15th interview; pursuant to the study protocol, two additional interviews were undertaken to confirm saturation, resulting in a total of 17 participants in this research. Those who agreed to participate, but were not required to be interviewed, were informed and thanked for their willingness to be involved in this study.

The demographic characteristics of those interviewed are presented in Table 1. For the purposes of this study, their professional role/designation/degree were not considered important variables of interest, since all the participants were primary care professionals. The objective of the study was not to compare the attitudes or behaviours of physicians vs. nurses, but instead to examine primary care professionals who may simply have different jobs or roles. In the context of the mitigation of climate-related health-care-associated harms, professional designation was not deemed an important variable of interest, since the primary care setting within which each professional practiced was broadly similar.

The constant comparative data analysis method utilized generated multiple categories, codes, and themes that were subsequently refined through consensus by the research team. Ultimately, three major themes were identified, as presented below:

Theme 1: There was some general awareness that some cleanliness practices are hygiene theatre, but there was limited time or interest on the part of the professionals to do anything more.
Theme 2: Climate impacts and potential climate mitigation opportunities of hygiene theatre were rarely considered “top-of-mind” priorities by the participants.
Theme 3: The participants believed that top-down interventions would be required to address hygiene theatre practices and achieve climate mitigation objectives.

Theme 1: There was some general awareness that some cleanliness practices are hygiene theatre, but there was limited time or interest on the part of the professionals to do anything more.

“Of course, I’ve always wondered—well, I guess, sorta known—well, it’s ridiculous to believe that those alcohol swabs we use in vaccinations actually do anything. But it does make patients feel good, feel positive, makes it seem clean, and if that encourages people to get vaccines, then I’m all for continuing to use them”.

“Those disinfectant wipes—they’re everywhere, I have no idea how much we must spend on them. But they are convenient, and—you know, with that nice lemony fresh smell—it’s not just patients, it’s all of us, it just feels so much cleaner and sanitary. And if that inspires confidence, makes us believe it’s safer, I think that’s a fine enough reason to keep using them, right?”

“Sure it might be what you call “theatre” or a performance, but really, isn’t that what all of heath care, especially primary care is in the end? Why do some people wear lab coats, why do we walk around with stethescopes around our necks? This is all just part of the show.”

“I’m not so sure. In the end, isn’t it simply better to err on the side of overabundant caution rather than risk it? These things are pretty harmless, pretty innocuous, so why not use them, just in case—in one particular situation—it actually is helpful.”

The participants in this study demonstrated some awareness of the questionable value associated with the six hygiene practices that were the focus of the interview. Only three of the 17 participants had heard the term “hygiene theatre” before the interview; all of the participants expressed immediate understanding of the term and its implications when it was presented. All of the participants described their own ambivalence around the practices that were the focus of the study, in particular interventions such as plexiglass shields, temperature checks, and the use of hand and surface sanitizing solutions and wipes. All of the participants expressed a similar belief that the convenience and time-saving features of these interventions were significant drivers of adoption in primary care. For example, while all of the participants believed that hand washing with soap and water was likely equally effective and more cost-effective than commercial hand sanitizers, they noted that a limited availability of sinks, the time required to dry wet hands, and other inconveniences simply made it faster and easier to rely on chemical alternatives in a busy primary care practice. Many participants noted that, following the COVID-19 pandemic, hand-sanitizing solutions were now routinely expected by patients, some of whom became alarmed if they saw their professionals washing hands rather than using solutions out of a bottle.

The power of inertia in driving cleaning practices was discussed frequently: having invested in equipment and supplies during the COVID-19 pandemic and having become accustomed to those routines and protocols, the participants in this study indicated that it was difficult to change behaviours—and frankly, not worth the time and effort to do so given all the other priorities and time pressures faced by busy primary care providers. As a result, despite some awareness of limited evidence regarding their value, hygiene practices perpetuate over time and are amplified as new trainees and students are mentored into primary care, assuming that the practices they observe are all essential and useful.

Most participants, when prompted to reflect further, understood the questionable value of the practices described, but also adopted a post hoc “if it’s not broken, don’t fix it” attitude. While the hygiene practices themselves may be of questionable value, they provide some form of psychological comfort for patients (and some professionals), and since they are not necessarily “harmful” to patients, there is no urgency or incentive to change them. The question of the climate-related harm associated with these practices did not arise spontaneously or naturally from the participants, nor were any financial harms or wastage issues discussed unless prompted by the interviewer.

The pervasiveness and tenacity of hygiene practices of unclear value or where mixed or inconclusive evidence exists regarding infection-prevention impacts were ultimately described in terms of the time and energy required to change something that was not framed as harmful or damaging for patients or professionals. Without that incentive to drive change, prioritizing changes and finding the time and bandwidth to do so are unlikely.

Theme 2: Climate impacts and potential climate mitigation opportunities of hygiene theatre were rarely considered “top-of-mind” priorities by the participants.

“Of course, we all know and worry about climate change. But in primary care there’s so much more, so many other urgent priorities, that unless it’s going to result in catastrophe tomorrow—well, it goes down at the bottom of the list. That’s kinda how I think most of us—well, me at least—that’s how I’d see it in terms of dealing with some of these questionable hygiene things”.

“How many vaccines, injections, needles have I given in my career? Thousands, at least. And each one has a disposable needle, syringe, and those swabs. How much landfill have I generated? How much garbage? How much could have been diverted if—I don’t know—we knew more, or thought a bit more about it? But that’s the problem right? Who has time, who has bandwidth to learn about this with all the other stuff we need to keep up on? Who has time to think about this when it’s like you’re barely keeping your head above water with primary care?”

“Getting this on our radar in primary care, that’s the challenge I think, right? I mean once you start talking about it—well it’s pretty obvious we all know most of the stuff we do around cleaning and stuff, it’s just for show. But that is important, patients expect it and well, it’s part of our routines now too. I wish I knew how to get this on the radar because, well, if it was, I think [primary care health care providers] would be able to move pretty quickly to change and reduce and remove things that have no evidence they work.”

“I wonder though, is it even worth it to worry about this? We have so much else on our plate, so much else to do, I don’t think this will ever get traction.”

All of the participants in this study noted the stress and time demands placed on primary-care health care providers, including the necessity for continuous professional development and learning associated with evolving medical sciences. The participants spoke about personal and professional struggles associated with the prioritization of critical issues and the seeming impossibility of being able to stay up to date on every new and important idea or development. In this context, the climate conscious practice and awareness of climate change mitigation strategies was viewed by the participants as yet “one more thing” to add to an already overburdened set of priorities. Despite good intentions and understanding and willingness to recognize the pending catastrophe associated with climate change, the longer-term time horizons associated with experiencing immediate impacts de-prioritizes action on climate change compared with other primary care priorities. Thus, presenting changes to unhelpful and unnecessary hygiene theatre practices as a climate mitigation strategy for primary care did not resonate with the participants, most of whom believed that the time, energy, and work required to overcome established and routinized practices, and the inertia inherent in hygiene therapy, would be thought to be too high a cost by most primary care providers if left to their own devices.

Every participant in this study fully acknowledged and accepted the realities of climate science and most indicated that, in their personal lives, they had embraced opportunities for climate change mitigation through (for example) opting for public transit rather than driving, or recycling, or choosing consumer products with minimal packaging. In their professional lives, however, the participants reported challenges in embracing climate-conscious practice due to an absence of centralized guidance or top-down regulation that told them what to do. They described the challenges associated with determining for themselves how to mitigate and practice in a climate-conscious way when they were already so overburdened with other priorities and day-to-day tasks in primary care.

Many participants highlighted an alternative framing of the issue of hygiene theatre: the idea that is unnecessarily costly and consumes precious financial resources within primary care. Arguing for a reduction in hygiene theatre as a cost-saving rather than climate change mitigation strategy might, in some circumstances, accelerate critical reflection and increase interest, particularly in practices faced with financial challenges. These participants noted that primary care practices are constantly adjusting their work and priorities based on budgetary constraints, and the costs associated with hygiene theatre could potentially be a more expeditious way of changing practices than arguing from a climate change perspective, while still achieving the same objective.

Theme 3: The participants believed that top-down interventions would be required to address hygiene theatre practices and achieve climate mitigation objectives.

“I would personally love it if someone just told us—made us—stop using disposable hand wipes or [unit of use disposable] blister packs for medications. Of course, we know—who doesn’t—how wasteful that all is. But no one is going to change unless someone tells them, forces them to. So—if the [licensing bodies] or [professional associations] issued rules staying “thou shall not unnecessarily check temperatures” or unnecessarily supposedly deep clean—well then, it would stop. And none of us would feel guilty about, “what if actually this time I really should have checked that temperature?”

“It’s kinda unfair I think to put the burden of all this on the already overburdened [primary care provider]. We just don’t have the bandwidth or the time, honestly, to figure this out. It would be great if, I don’t know, my union, or the employers, or I don’t know someone who is in charge gave us the permission to do away with these useless activities. Until then—you know, the fear of being sued, liability, being negligent—all of that is going to make us keep on doing what we have always been doing, unless somebody bigger and more powerful gives the okay to stop doing it.”

“What I don’t understand—you know, being on top of this evidence, this data—why haven’t the [professional associations] or [ licensing bodies] or people in charge—I mean if they know this, just tell us to stop doing these useful things. That’s it. That’s all they need to do. We are very used to, I mean we like following rules and algorithms and guidelines That’s why we do things like alcohol swabs or temperature checks—because at some point it ended up in a flow chart. If that doesn’t make sense any more or it isn’t needed—just take it out and we will instantly be able to stop hygiene theatre.”

There was consensus amongst all of the participants that the translation of evidence associated with unnecessary hygiene theatre into practice guidelines and algorithms would quickly result in the cessation of unnecessary practices. Rather than have individual clinicians find the time and energy to research for themselves and undertake local, practice-specific modifications to existing protocols, guidance from experts, professional associations, unions, and licensing bodies would be a far more effective and efficient way to ensure that hygiene theatre ceases. Further, this approach would reduce the likelihood that individual clinicians would feel guilty or concerned about their potential liability in going against current hygiene theatre algorithms or established practices.

The top-down approach to addressing this issue would not need to be framed in terms of climate change mitigation, but simply in terms of best evidence with respect to quality, efficacy and safety. The argument against hygiene theatre measures is not simply about the money it costs or the garbage it generates, but, from a primary care clinician’s perspective, about the fact that it takes time and does not actually do what it is purported to accomplish—make patients safer and reduce the risk of transmission of infectious diseases. If sufficient evidence exists to make this case, that is the primary concern and interest of primary care providers and should be sufficient to trigger changes in, for example, vaccination protocols that are universally followed.

Several participants raised the question that, if this change in guidelines/algorithms has not already happened, does this potentially indicate that there is insufficient evidence to conclude that hygiene theatre practices are in fact unhelpful and unnecessary? They noted how complex practice changes and knowledge translation can be, but also agreed that expecting busy individual clinicians to manage this without top-down intervention was unrealistic.

## 4. Discussion

The environmental impact of unquestioned adherence to hygiene theatre protocols may be difficult to quantify, but could be significant. Data during the COVID-19 pandemic suggest that more than 175 million alcohol swabs were purchased by the Canadian government and provincial counterparts to support the mass vaccination campaign [15]. Each of these swabs, and the foil packet in which they were delivered, was treated as medical waste and incinerated or put into secured landfills rather than recycled—or completely avoided in the first place, as the value of alcohol swabs as an infection reduction technique is unclear. This one example highlights the potential value of examining a suite of hygiene protocols and techniques that may be unnecessary, costly, time consuming, and unhelpful, and that may add to the carbon footprint of health care delivery.

This study highlights the challenges associated with translating evidence (or in this case, the lack of clear evidence to support a practice) into clinical change, particularly in a busy and overburdened primary care setting. The power of inertia in perpetuating clinical practices could be significant. As the participants in this study noted, it is unrealistic to expect individual busy practitioners to undertake their own research, deliberate on their own, and implement practice-level changes by themselves. Despite awareness and acceptance of the harms and risks associated with climate change, recognition that inconclusive or mixed evidence exists with respect to some commonly used infection control practices, and willingness to explore other options, the participants in this study noted how burdensome it would be to expect front-line clinicians to do this analysis themselves. Instead, there was strong consensus that a top-down approach to practice change that begins with changes in the guidance and algorithms used by clinicians to guide their work would be needed. They noted that clinicians going against established practice or skipping steps in established flow charts is unlikely: they might feel guilty as a rule-breaker or fear legal/liability consequences for not doing what everyone else is doing or what the existing guidelines recommend. In the context of hygiene theatre activities, the sense that such behaviour does not harm patients and may in many cases provide some psychological comfort or reassurance further explains the reluctance to independently change the existing practices.

The climate mitigation opportunities presented by addressing hygiene theatre in health care could be significant, given the scale and ubiquity of these practices in day-to-day health care work. The findings from this study, however, suggest that reliance on front-line practitioners to initiate changes may be unlikely given the clinical realities they face. Instead, a top-down approach in which experts and those with positional powers (such as licensing bodies, employers, unions, and professional associations) generate the clinical guidance, algorithms and flow-charts necessary to drive practice change was viewed as essential. Even with this top-down direction, the participants in this study noted the “stubbornness” of inertia in primary care: once processes and protocols have been established in practice, there is great difficulty in breaking old routines. As a result, hygiene theatre, particularly activities that emerged in the early days of the COVID-19 pandemic, continue to perpetuate.

The climate consequences of health care work are significant and the carbon footprint of primary care professionals is a cause for concern: in doing work that is aimed at making people well, health care professionals are polluting and contaminating our world in ways that are actually exacerbating the very illnesses (such as asthma) that they are working hard to treat [16]. This irony points to the need to address the issue. Addressing hygiene theatre seems to be a reasonable way of reducing carbon footprints (along with reducing the risks of antimicrobial resistance and environmental contamination): these performative activities have minimal or no clear evidence of clinical efficacy or value, are operational rather than medical in their orientation, and mainly emerged in the last half-decade in response to a once-in-a-generation pandemic. The tenacity of these practices and difficulties associated with dislodging them once they are established highlight the challenges associated with climate mitigation work in primary care. Coalitions of regulators, educators, employers, unions and professional associations, spurred on by concerned practitioners, have the potential to use their clout to change guidance documents and practice algorithms in a positive way.

This study has several strengths. It was amongst the first to examine the relatively recent phenomenon of hygiene theatre from the perspective of climate change mitigation. It focused on primary care, the largest, and most impactful, part of the health care system that touches the lives of virtually every citizen. It gave voice to the experiences of primary care clinicians, those physicians, nurses, nurse practitioners and pharmacists at the heart of the primary care system, who are in the best position to operationalize needed changes. It also highlighted the challenge of practice change, but provided some interesting and practical options to consider, including coalition building among leaders in the profession, and reframing the issue in terms of financial encumbrance and/or a lack of clinical evidence as a way of driving forward change that is ultimately (if not explicitly) climate-friendly. The research method was built around the COREQ checklist to enhance the quality and indicativeness. Techniques such as independent coding by reviewers, member checking by the participants, and the triangulation of data analyses were undertaken to ensure rigour and quality in the research process.

There are also limitations. This was a small, exploratory, qualitative study undertaken in one jurisdiction (Ontario, Canada), so it may not be generalizable beyond there. The recruitment of participants was undertaken primarily for convenience and therefore cannot be viewed as representative of that jurisdiction. Without funding, it was not possible to compensate the participants for their time, meaning that those who eventually chose to participate may have already had pre-existing beliefs and perspectives on this research topic that may not be representative of the broader population of health professionals. Selection bias may be an issue that could have influenced the results, particularly since convenience sampling within professional networks was the primary vehicle used to recruit the participants. The semi-structured interview format may also be a limitation: while it was selected in order to facilitate more personalized conversations and information gathering, it may have skewed the conversations in a manner that was leading for some participants who may have detected the researchers’ bias through the conversation and simply agreed. While semi-structured interview prompts were designed to prompt discussion and reflection on clinical practice, there is some risk that in simply focusing attention on a specific practice (such as hand sanitizing), the participants may have interpreted this as a signal that the practice itself was being questioned. Further, the decision to not differentiate findings based on the professional designation of the participant may be a limitation. For example, pharmacists may have more insights into the use of unit-of-use blister packaging than family physicians, and therefore, they may have provided different unique insights. The decision to not stratify the participants based on their professional designation was made for pragmatic reasons, as a way of managing the limited research resources that were available while still allowing us to achieve thematic saturation as an endpoint of the research. As a result of these limitations, caution in generalizing the findings from this study to other contexts is warranted.

This study was a first step, and as such, it represents small-scale exploratory research that points to potential future studies that could help us better understand how to reduce hygiene theatre in meaningful ways. Future research may involve those working in regulatory bodies or professional associations who may have greater power to change the existing practice guidelines and algorithms and influence practice changes across health systems. Further work exploring how knowledge translation in this context could be mobilized and expanded is also necessary in order to drive forward practice change. In addition, profession-specific research focused on pharmacists, family physicians, nurses and nurse practitioners may provide valuable insights.

## 5. Conclusions

The climate impact of health care work can be significant and negative, and unnecessary and unhelpful hygiene theatre measures may be one important, but overlooked, contributor to the overall carbon footprint of health care work. Though it may appear simple and self-evident to simply abandon practices for which mixed or inconclusive evidence of the infection-control value exists, this study highlights that the process is more complex than it may first appear. It is imperative to recognize the reality that those who are expected to change—front-line primary care providers—are already overburdened, under-resourced, and therefore lacking the time, energy or bandwidth to undertake changes to established routines on their own. Instead, building coalitions of leaders in the field to drive and support practice change will likely be necessary in order to support those front-line practitioners in achieving climate change mitigation objectives.

## Figures and Tables

**Table 1:** Demographic characteristics of research participants (n = 17).

**Sex**	Male = 7; female = 10
**Age**	Mean = 44.9 years old (range = 29 to 66 years old, stand. dev. = 6.7 years)
**Profession**	Physician (MD or equivalent): 4 Nurse (RN or equivalent): 6 Pharmacist (Pharm D or BScPhm or equivalent): 4 Nurse practitioner/advanced practice nurse: 3
**Geographic Location**	Large urban area: 8 Mid-sized city: 2 Suburban: 4 Small town/rural: 3
**Public Health or Infect**	Yes = 1; no = 16

## Data Availability

The data presented in this study are available from the corresponding author upon request, in order to protect the confidentiality of the study participants.
